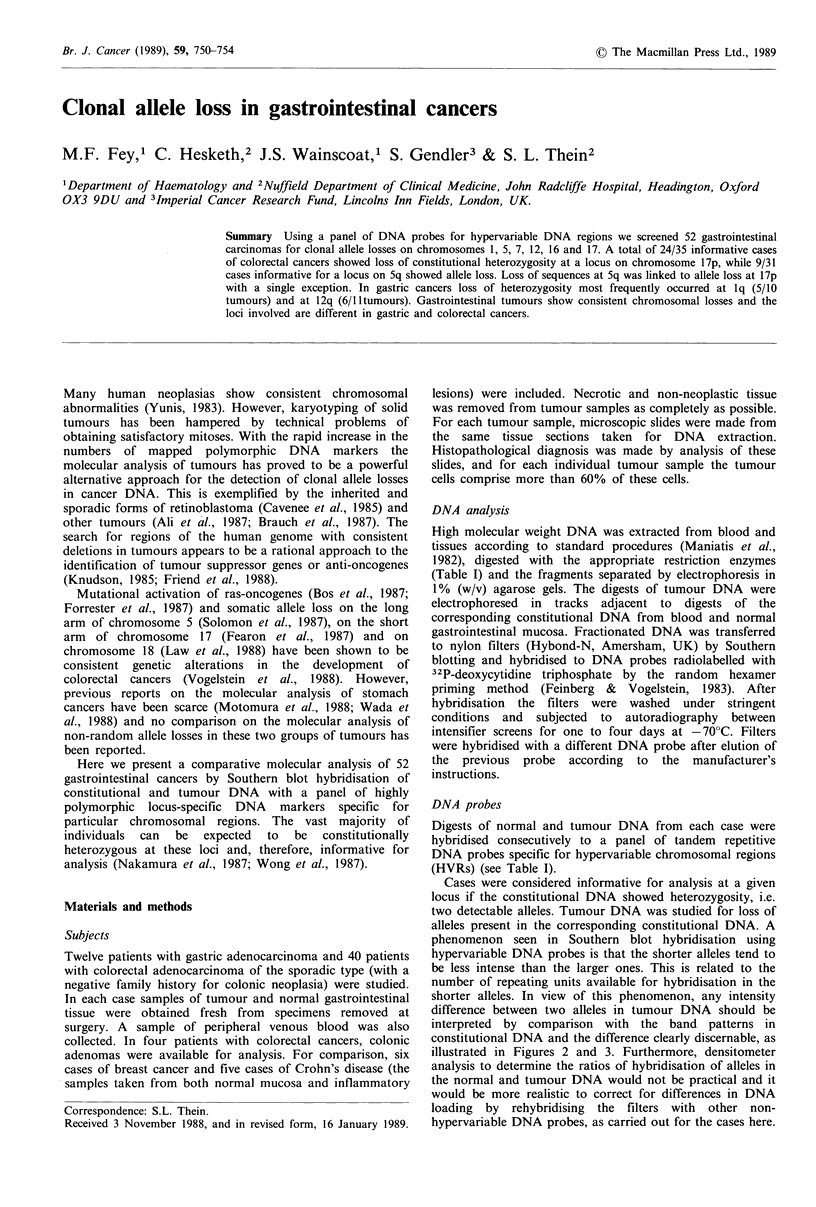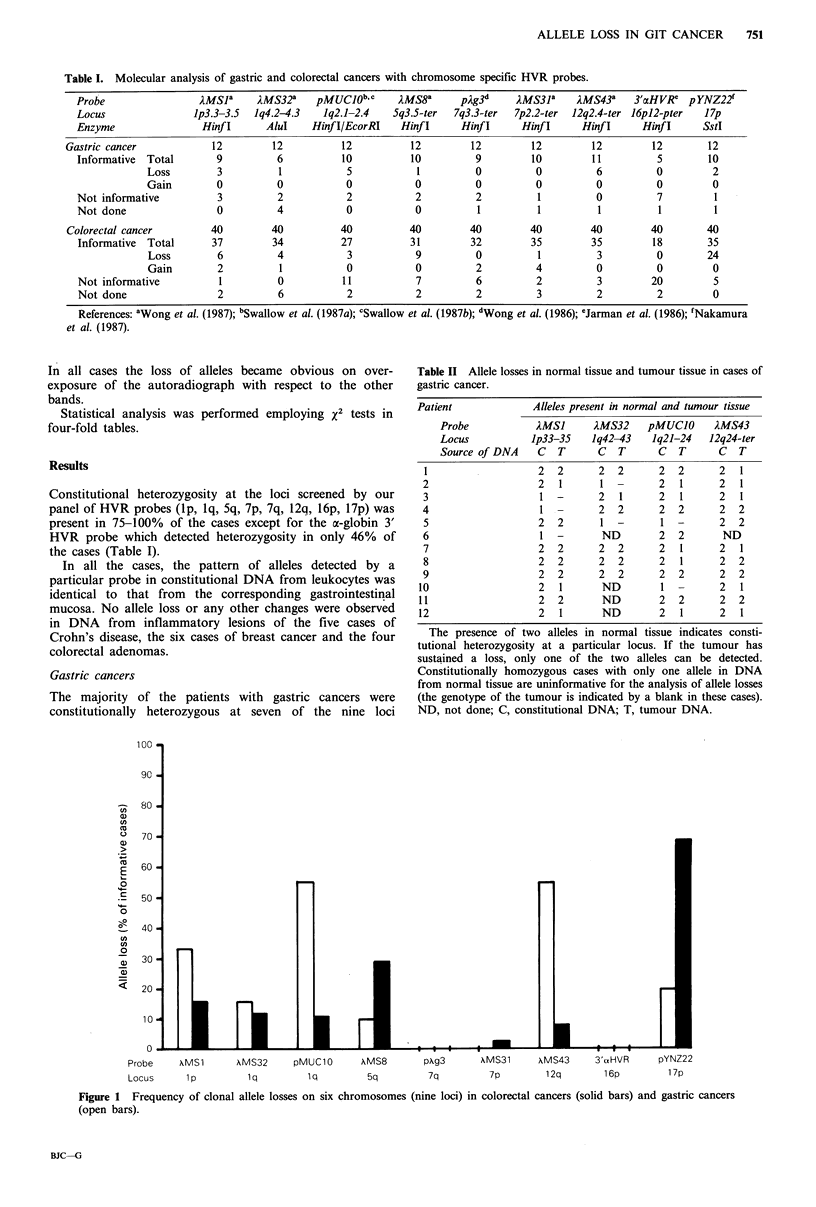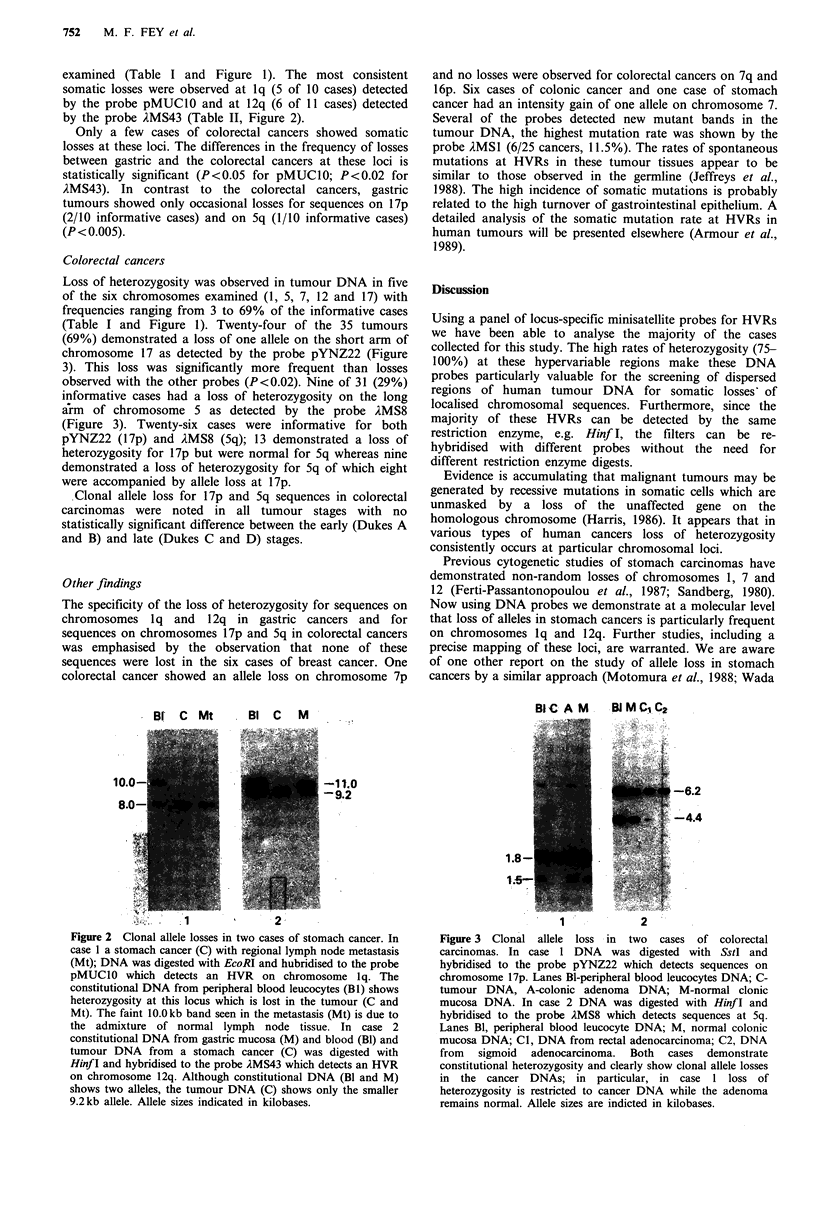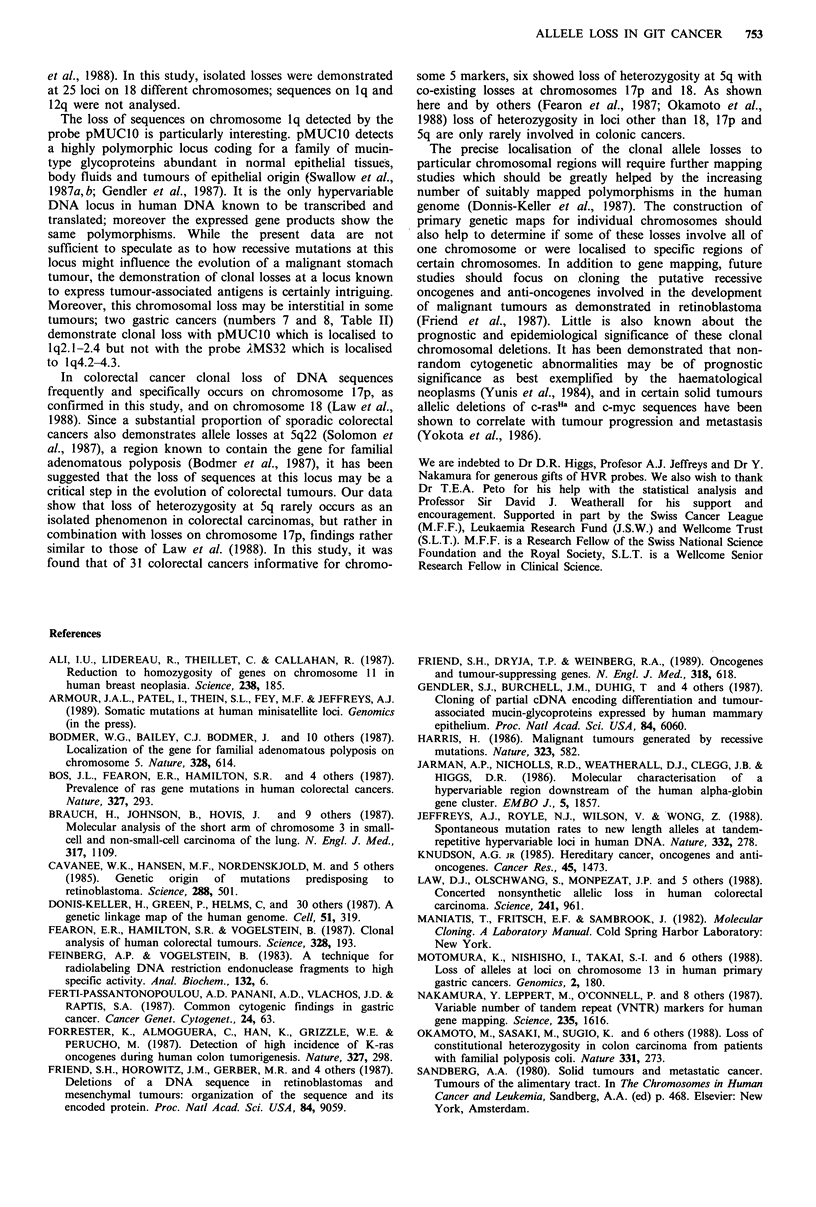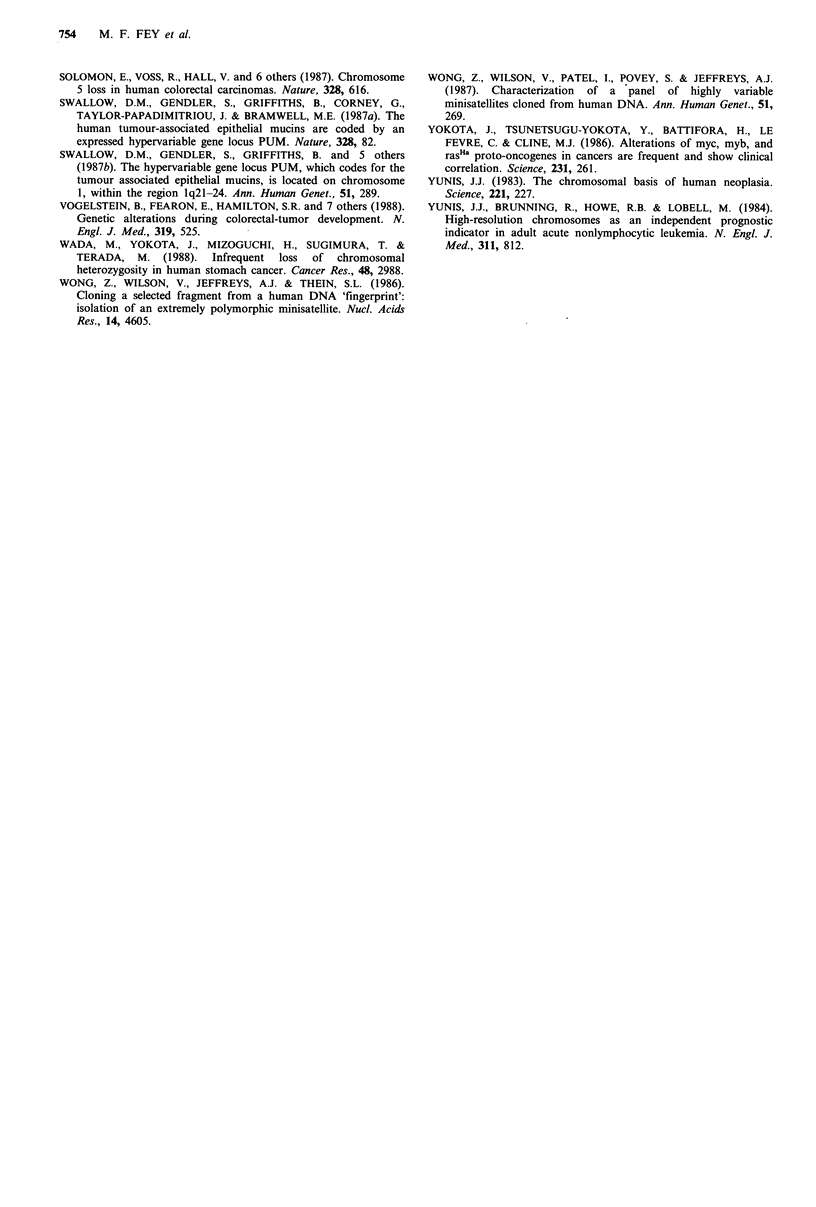# Clonal allele loss in gastrointestinal cancers.

**DOI:** 10.1038/bjc.1989.157

**Published:** 1989-05

**Authors:** M. F. Fey, C. Hesketh, J. S. Wainscoat, S. Gendler, S. L. Thein

**Affiliations:** Department of Haematology, John Radcliffe Hospital, Headington, Oxford, UK.

## Abstract

**Images:**


*Br. J. Cancer* (1989), **59**, 750–754 © The Macmillan Press Ltd., 1989

### Clonal allele loss in gastrointestinal cancers

M.F. Fey,[1] C. Hesketh,[2] J.S. Wainscoat,[1] S. Gendler[3] & S. L. Thein[2]

[1]*Department of Haematology and* [2]*Nuffield Department of Clinical Medicine, John Radcliffe Hospital, Headington, Oxford OX3 9DU and* [3]*Imperial Cancer Research Fund, Lincolns Inn Fields, London, UK.*

**Summary** Using a panel of DNA probes for hypervariable DNA regions we screened 52 gastrointestinal carcinomas for clonal allele losses on chromosomes 1, 5, 7, 12, 16 and 17. A total of 24/35 informative cases of colorectal cancers showed loss of constitutional heterozygosity at a locus on chromosome 17p, while 9/31 cases informative for a locus on 5q showed allele loss. Loss of sequences at 5q was linked to allele loss at 17p with a single exception. In gastric cancers loss of heterozygosity most frequently occurred at 1q (5/10 tumours) and at 12q (6/11tumours). Gastrointestinal tumours show consistent chromosomal losses and the loci involved are different in gastric and colorectal cancers.

Many human neoplasias show consistent chromosomal abnormalities (Yunis, 1983). However, karyotyping of solid tumours has been hampered by technical problems of obtaining satisfactory mitoses. With the rapid increase in the numbers of mapped polymorphic DNA markers the molecular analysis of tumours has proved to be a powerful alternative approach for the detection of clonal allele losses in cancer DNA. This is exemplified by the inherited and sporadic forms of retinoblastoma (Cavenee *et al*., 1985) and other tumours (Ali *et al*., 1987; Brauch *et al*., 1987). The search for regions of the human genome with consistent deletions in tumours appears to be a rational approach to the identification of tumour suppressor genes or anti-oncogenes (Knudson, 1985; Friend *et al*., 1988).

Mutational activation of ras-oncogenes (Bos *et al*., 1987; Forrester *et al*., 1987) and somatic allele loss on the long arm of chromosome 5 (Solomon *et al*., 1987), on the short arm of chromosome 17 (Fearon *et al*., 1987) and on chromosome 18 (Law *et al*., 1988) have been shown to be consistent genetic alterations in the development of colorectal cancers (Vogelstein *et al*., 1988). However, previous reports on the molecular analysis of stomach cancers have been scarce (Motomura *et al*., 1988; Wada *et al*., 1988) and no comparison on the molecular analysis of non-random allele losses in these two groups of tumours has been reported.

Here we present a comparative molecular analysis of 52 gastrointestinal cancers by Southern blot hybridisation of constitutional and tumour DNA with a panel of highly polymorphic locus-specific DNA markers specific for particular chromosomal regions. The vast majority of individuals can be expected to be constitutionally heterozygous at these loci and, therefore, informative for analysis (Nakamura *et al*., 1987; Wong *et al*., 1987).

### Materials and methods

### *Subjects*

Twelve patients with gastric adenocarcinoma and 40 patients with colorectal adenocarcinoma of the sporadic type (with a negative family history for colonic neoplasia) were studied. In each case samples of tumour and normal gastrointestinal tissue were obtained fresh from specimens removed at surgery. A sample of peripheral venous blood was also collected. In four patients with colorectal cancers, colonic adenomas were available for analysis. For comparison, six cases of breast cancer and five cases of Crohn's disease (the samples taken from both normal mucosa and inflammatory lesions) were included. Necrotic and non-neoplastic tissue was removed from tumour samples as completely as possible. For each tumour sample, microscopic slides were made from the same tissue sections taken for DNA extraction. Histopathological diagnosis was made by analysis of these slides, and for each individual tumour sample the tumour cells comprise more than 60% of these cells.

Correspondence: S.L. Thein.
Received 3 November 1988, and in revised form, 16 January 1989.

### *DNA analysis*

High molecular weight DNA was extracted from blood and tissues according to standard procedures (Maniatis *et al*., 1982), digested with the appropriate restriction enzymes (Table I) and the fragments separated by electrophoresis in 1% (w/v) agarose gels. The digests of tumour DNA were electrophoresed in tracks adjacent to digests of the corresponding constitutional DNA from blood and normal gastrointestinal mucosa. Fractionated DNA was transferred to nylon filters (Hybond-N, Amersham, UK) by Southern blotting and hybridised to DNA probes radiolabelled with $^{32}$P-deoxycytidine triphosphate by the random hexamer priming method (Feinberg & Vogelstein, 1983). After hybridisation the filters were washed under stringent conditions and subjected to autoradiography between intensifier screens for one to four days at $-70°$C. Filters were hybridised with a different DNA probe after elution of the previous probe according to the manufacturer's instructions.

### *DNA probes*

Digests of normal and tumour DNA from each case were hybridised consecutively to a panel of tandem repetitive DNA probes specific for hypervariable chromosomal regions (HVRs) (see Table I).

Cases were considered informative for analysis at a given locus if the constitutional DNA showed heterozygosity, i.e. two detectable alleles. Tumour DNA was studied for loss of alleles present in the corresponding constitutional DNA. A phenomenon seen in Southern blot hybridisation using hypervariable DNA probes is that the shorter alleles tend to be less intense than the larger ones. This is related to the number of repeating units available for hybridisation in the shorter alleles. In view of this phenomenon, any intensity difference between two alleles in tumour DNA should be interpreted by comparison with the band patterns in constitutional DNA and the difference clearly discernable, as illustrated in Figures 2 and 3. Furthermore, densitometer analysis to determine the ratios of hybridisation of alleles in the normal and tumour DNA would not be practical and it would be more realistic to correct for differences in DNA loading by rehybridising the filters with other non-hypervariable DNA probes, as carried out for the cases here.

**Table I.** Molecular analysis of gastric and colorectal cancers with chromosome specific HVR probes.

| *Probe* | λMS1[a] | λMS32[a] | pMUC10[b,c] | λMS8[a] | pλg3[d] | λMS31[a] | λMS43[a] | 3′αHVR[e] | pYNZ22[f] |
|---|---|---|---|---|---|---|---|---|---|
| *Locus* | *1p3.3–3.5* | *1q4.2–4.3* | *1q2.1–2.4* | *5q3.5-ter* | *7q3.3-ter* | *7p2.2-ter* | *12q2.4-ter* | *16p12-pter* | *17p* |
| *Enzyme* | *Hinf*I | *Alu*I | *Hinf*I/*Ecor*RI | *Hinf*I | *Hinf*I | *Hinf*I | *Hinf*I | *Hinf*I | *Sst*I |
| *Gastric cancer* | 12 | 12 | 12 | 12 | 12 | 12 | 12 | 12 | 12 |
| Informative Total | 9 | 6 | 10 | 10 | 9 | 10 | 11 | 5 | 10 |
| Loss | 3 | 1 | 5 | 1 | 0 | 0 | 6 | 0 | 2 |
| Gain | 0 | 0 | 0 | 0 | 0 | 0 | 0 | 0 | 0 |
| Not informative | 3 | 2 | 2 | 2 | 2 | 1 | 0 | 7 | 1 |
| Not done | 0 | 4 | 0 | 0 | 1 | 1 | 1 | 1 | 1 |
| *Colorectal cancer* | 40 | 40 | 40 | 40 | 40 | 40 | 40 | 40 | 40 |
| Informative Total | 37 | 34 | 27 | 31 | 32 | 35 | 35 | 18 | 35 |
| Loss | 6 | 4 | 3 | 9 | 0 | 1 | 3 | 0 | 24 |
| Gain | 2 | 1 | 0 | 0 | 2 | 4 | 0 | 0 | 0 |
| Not informative | 1 | 0 | 11 | 7 | 6 | 2 | 3 | 20 | 5 |
| Not done | 2 | 6 | 2 | 2 | 2 | 3 | 2 | 2 | 0 |

References: [a]Wong *et al.* (1987); [b]Swallow *et al.* (1987*a*); [c]Swallow *et al.* (1987*b*); [d]Wong *et al.* (1986); [e]Jarman *et al.* (1986); [f]Nakamura *et al.* (1987).

In all cases the loss of alleles became obvious on over-exposure of the autoradiograph with respect to the other bands.

Statistical analysis was performed employing $\chi^2$ tests in four-fold tables.

### Results

Constitutional heterozygosity at the loci screened by our panel of HVR probes (1p, 1q, 5q, 7p, 7q, 12q, 16p, 17p) was present in 75–100% of the cases except for the α-globin 3′ HVR probe which detected heterozygosity in only 46% of the cases (Table I).

In all the cases, the pattern of alleles detected by a particular probe in constitutional DNA from leukocytes was identical to that from the corresponding gastrointestinal mucosa. No allele loss or any other changes were observed in DNA from inflammatory lesions of the five cases of Crohn's disease, the six cases of breast cancer and the four colorectal adenomas.

### *Gastric cancers*

The majority of the patients with gastric cancers were constitutionally heterozygous at seven of the nine loci

**Table II** Allele losses in normal tissue and tumour tissue in cases of gastric cancer.

| *Patient* | *Alleles present in normal and tumour tissue* | | | | | | | |
|---|---|---|---|---|---|---|---|---|
| *Probe* | *λMS1* | | *λMS32* | | *pMUC10* | | *λMS43* | |
| *Locus* | *1p33–35* | | *1q42–43* | | *1q21–24* | | *12q24-ter* | |
| *Source of DNA* | *C* | *T* | *C* | *T* | *C* | *T* | *C* | *T* |
| 1 | 2 | 2 | 2 | 2 | 2 | 2 | 2 | 1 |
| 2 | 2 | 1 | 1 | – | 2 | 1 | 2 | 1 |
| 3 | 1 | – | 2 | 1 | 2 | 1 | 2 | 1 |
| 4 | 1 | – | 2 | 2 | 2 | 2 | 2 | 2 |
| 5 | 2 | 2 | 1 | – | 1 | – | 2 | 2 |
| 6 | 1 | – | ND | | 2 | 2 | ND | |
| 7 | 2 | 2 | 2 | 2 | 2 | 1 | 2 | 1 |
| 8 | 2 | 2 | 2 | 2 | 2 | 1 | 2 | 2 |
| 9 | 2 | 2 | 2 | 2 | 2 | 2 | 2 | 2 |
| 10 | 2 | 1 | ND | | 1 | – | 2 | 1 |
| 11 | 2 | 2 | ND | | 2 | 2 | 2 | 2 |
| 12 | 2 | 1 | ND | | 2 | 1 | 2 | 1 |

The presence of two alleles in normal tissue indicates constitutional heterozygosity at a particular locus. If the tumour has sustained a loss, only one of the two alleles can be detected. Constitutionally homozygous cases with only one allele in DNA from normal tissue are uninformative for the analysis of allele losses (the genotype of the tumour is indicated by a blank in these cases). ND, not done; C, constitutional DNA; T, tumour DNA.

**Figure 1** Frequency of clonal allele losses on six chromosomes (nine loci) in colorectal cancers (solid bars) and gastric cancers (open bars).

examined (Table I and Figure 1). The most consistent somatic losses were observed at 1q (5 of 10 cases) detected by the probe pMUC10 and at 12q (6 of 11 cases) detected by the probe λMS43 (Table II, Figure 2).

Only a few cases of colorectal cancers showed somatic losses at these loci. The differences in the frequency of losses between gastric and the colorectal cancers at these loci is statistically significant ($P<0.05$ for pMUC10; $P<0.02$ for λMS43). In contrast to the colorectal cancers, gastric tumours showed only occasional losses for sequences on 17p (2/10 informative cases) and on 5q (1/10 informative cases) ($P<0.005$).

*Colorectal cancers*

Loss of heterozygosity was observed in tumour DNA in five of the six chromosomes examined (1, 5, 7, 12 and 17) with frequencies ranging from 3 to 69% of the informative cases (Table I and Figure 1). Twenty-four of the 35 tumours (69%) demonstrated a loss of one allele on the short arm of chromosome 17 as detected by the probe pYNZ22 (Figure 3). This loss was significantly more frequent than losses observed with the other probes ($P<0.02$). Nine of 31 (29%) informative cases had a loss of heterozygosity on the long arm of chromosome 5 as detected by the probe λMS8 (Figure 3). Twenty-six cases were informative for both pYNZ22 (17p) and λMS8 (5q); 13 demonstrated a loss of heterozygosity for 17p but were normal for 5q whereas nine demonstrated a loss of heterozygosity for 5q of which eight were accompanied by allele loss at 17p.

Clonal allele loss for 17p and 5q sequences in colorectal carcinomas were noted in all tumour stages with no statistically significant difference between the early (Dukes A and B) and late (Dukes C and D) stages.

*Other findings*

The specificity of the loss of heterozygosity for sequences on chromosomes 1q and 12q in gastric cancers and for sequences on chromosomes 17p and 5q in colorectal cancers was emphasised by the observation that none of these sequences were lost in the six cases of breast cancer. One colorectal cancer showed an allele loss on chromosome 7p and no losses were observed for colorectal cancers on 7q and 16p. Six cases of colonic cancer and one case of stomach cancer had an intensity gain of one allele on chromosome 7. Several of the probes detected new mutant bands in the tumour DNA, the highest mutation rate was shown by the probe λMS1 (6/25 cancers, 11.5%). The rates of spontaneous mutations at HVRs in these tumour tissues appear to be similar to those observed in the germline (Jeffreys *et al.*, 1988). The high incidence of somatic mutations is probably related to the high turnover of gastrointestinal epithelium. A detailed analysis of the somatic mutation rate at HVRs in human tumours will be presented elsewhere (Armour *et al.*, 1989).

### Discussion

Using a panel of locus-specific minisatellite probes for HVRs we have been able to analyse the majority of the cases collected for this study. The high rates of heterozygosity (75–100%) at these hypervariable regions make these DNA probes particularly valuable for the screening of dispersed regions of human tumour DNA for somatic losses of localised chromosomal sequences. Furthermore, since the majority of these HVRs can be detected by the same restriction enzyme, e.g. *Hinf* I, the filters can be re-hybridised with different probes without the need for different restriction enzyme digests.

Evidence is accumulating that malignant tumours may be generated by recessive mutations in somatic cells which are unmasked by a loss of the unaffected gene on the homologous chromosome (Harris, 1986). It appears that in various types of human cancers loss of heterozygosity consistently occurs at particular chromosomal loci.

Previous cytogenetic studies of stomach carcinomas have demonstrated non-random losses of chromosomes 1, 7 and 12 (Ferti-Passantonopoulou *et al.*, 1987; Sandberg, 1980). Now using DNA probes we demonstrate at a molecular level that loss of alleles in stomach cancers is particularly frequent on chromosomes 1q and 12q. Further studies, including a precise mapping of these loci, are warranted. We are aware of one other report on the study of allele loss in stomach cancers by a similar approach (Motomura *et al.*, 1988; Wada

**Figure 2** Clonal allele losses in two cases of stomach cancer. In case 1 a stomach cancer (C) with regional lymph node metastasis (Mt); DNA was digested with *EcoRI* and hubridised to the probe pMUC10 which detects an HVR on chromosome 1q. The constitutional DNA from peripheral blood leucocytes (B1) shows heterozygosity at this locus which is lost in the tumour (C and Mt). The faint 10.0 kb band seen in the metastasis (Mt) is due to the admixture of normal lymph node tissue. In case 2 constitutional DNA from gastric mucosa (M) and blood (Bl) and tumour DNA from a stomach cancer (C) was digested with *Hinf*I and hybridised to the probe λMS43 which detects an HVR on chromosome 12q. Although constitutional DNA (Bl and M) shows two alleles, the tumour DNA (C) shows only the smaller 9.2 kb allele. Allele sizes indicated in kilobases.

**Figure 3** Clonal allele loss in two cases of colorectal carcinomas. In case 1 DNA was digested with *Sst*I and hybridised to the probe pYNZ22 which detects sequences on chromosome 17p. Lanes Bl-peripheral blood leucocytes DNA; C-tumour DNA, A-colonic adenoma DNA; M-normal clonic mucosa DNA. In case 2 DNA was digested with *Hinf*I and hybridised to the probe λMS8 which detects sequences at 5q. Lanes Bl, peripheral blood leucocyte DNA; M, normal colonic mucosa DNA; C1, DNA from rectal adenocarcinoma; C2, DNA from sigmoid adenocarcinoma. Both cases demonstrate constitutional heterozygosity and clearly show clonal allele losses in the cancer DNAs; in particular, in case 1 loss of heterozygosity is restricted to cancer DNA while the adenoma remains normal. Allele sizes are indicted in kilobases.

*et al.*, 1988). In this study, isolated losses were demonstrated at 25 loci on 18 different chromosomes; sequences on 1q and 12q were not analysed.

The loss of sequences on chromosome 1q detected by the probe pMUC10 is particularly interesting. pMUC10 detects a highly polymorphic locus coding for a family of mucin-type glycoproteins abundant in normal epithelial tissues, body fluids and tumours of epithelial origin (Swallow *et al.*, 1987*a*,*b*; Gendler *et al.*, 1987). It is the only hypervariable DNA locus in human DNA known to be transcribed and translated; moreover the expressed gene products show the same polymorphisms. While the present data are not sufficient to speculate as to how recessive mutations at this locus might influence the evolution of a malignant stomach tumour, the demonstration of clonal losses at a locus known to express tumour-associated antigens is certainly intriguing. Moreover, this chromosomal loss may be interstitial in some tumours; two gastric cancers (numbers 7 and 8, Table II) demonstrate clonal loss with pMUC10 which is localised to 1q2.1–2.4 but not with the probe λMS32 which is localised to 1q4.2–4.3.

In colorectal cancer clonal loss of DNA sequences frequently and specifically occurs on chromosome 17p, as confirmed in this study, and on chromosome 18 (Law *et al.*, 1988). Since a substantial proportion of sporadic colorectal cancers also demonstrates allele losses at 5q22 (Solomon *et al.*, 1987), a region known to contain the gene for familial adenomatous polyposis (Bodmer *et al.*, 1987), it has been suggested that the loss of sequences at this locus may be a critical step in the evolution of colorectal tumours. Our data show that loss of heterozygosity at 5q rarely occurs as an isolated phenomenon in colorectal carcinomas, but rather in combination with losses on chromosome 17p, findings rather similar to those of Law *et al.* (1988). In this study, it was found that of 31 colorectal cancers informative for chromosome 5 markers, six showed loss of heterozygosity at 5q with co-existing losses at chromosomes 17p and 18. As shown here and by others (Fearon *et al.*, 1987; Okamoto *et al.*, 1988) loss of heterozygosity in loci other than 18, 17p and 5q are only rarely involved in colonic cancers.

The precise localisation of the clonal allele losses to particular chromosomal regions will require further mapping studies which should be greatly helped by the increasing number of suitably mapped polymorphisms in the human genome (Donnis-Keller *et al.*, 1987). The construction of primary genetic maps for individual chromosomes should also help to determine if some of these losses involve all of one chromosome or were localised to specific regions of certain chromosomes. In addition to gene mapping, future studies should focus on cloning the putative recessive oncogenes and anti-oncogenes involved in the development of malignant tumours as demonstrated in retinoblastoma (Friend *et al.*, 1987). Little is also known about the prognostic and epidemiological significance of these clonal chromosomal deletions. It has been demonstrated that non-random cytogenetic abnormalities may be of prognostic significance as best exemplified by the haematological neoplasms (Yunis *et al.*, 1984), and in certain solid tumours allelic deletions of c-ras$^{Ha}$ and c-myc sequences have been shown to correlate with tumour progression and metastasis (Yokota *et al.*, 1986).

We are indebted to Dr D.R. Higgs, Profesor A.J. Jeffreys and Dr Y. Nakamura for generous gifts of HVR probes. We also wish to thank Dr T.E.A. Peto for his help with the statistical analysis and Professor Sir David J. Weatherall for his support and encouragement. Supported in part by the Swiss Cancer League (M.F.F.), Leukaemia Research Fund (J.S.W.) and Wellcome Trust (S.L.T.). M.F.F. is a Research Fellow of the Swiss National Science Foundation and the Royal Society, S.L.T. is a Wellcome Senior Research Fellow in Clinical Science.

### References

ALI, I.U., LIDEREAU, R., THEILLET, C. & CALLAHAN, R. (1987). Reduction to homozygosity of genes on chromosome 11 in human breast neoplasia. *Science*, **238**, 185.

ARMOUR, J.A.L., PATEL, I., THEIN, S.L., FEY, M.F. & JEFFREYS, A.J. (1989). Somatic mutations at human minisatellite loci. *Genomics* (in the press).

BODMER, W.G., BAILEY, C.J. BODMER, J. and 10 others (1987). Localization of the gene for familial adenomatous polyposis on chromosome 5. *Nature*, **328**, 614.

BOS, J.L., FEARON, E.R., HAMILTON, S.R. and 4 others (1987). Prevalence of ras gene mutations in human colorectal cancers. *Nature*, **327**, 293.

BRAUCH, H., JOHNSON, B., HOVIS, J. and 9 others (1987). Molecular analysis of the short arm of chromosome 3 in small-cell and non-small-cell carcinoma of the lung. *N. Engl. J. Med.*, **317**, 1109.

CAVANEE, W.K., HANSEN, M.F., NORDENSKJOLD, M. and 5 others (1985). Genetic origin of mutations predisposing to retinoblastoma. *Science*, **288**, 501.

DONIS-KELLER, H., GREEN, P., HELMS, C, and 30 others (1987). A genetic linkage map of the human genome. *Cell*, **51**, 319.

FEARON, E.R., HAMILTON, S.R. & VOGELSTEIN, B. (1987). Clonal analysis of human colorectal tumours. *Science*, **328**, 193.

FEINBERG, A.P. & VOGELSTEIN, B. (1983). A technique for radiolabeling DNA restriction endonuclease fragments to high specific activity. *Anal. Biochem.*, **132**, 6.

FERTI-PASSANTONOPOULOU, A.D. PANANI, A.D., VLACHOS, J.D. & RAPTIS, S.A. (1987). Common cytogenic findings in gastric cancer. *Cancer Genet. Cytogenet.*, **24**, 63.

FORRESTER, K., ALMOGUERA, C., HAN, K., GRIZZLE, W.E. & PERUCHO, M. (1987). Detection of high incidence of K-ras oncogenes during human colon tumorigenesis. *Nature*, **327**, 298.

FRIEND, S.H., HOROWITZ, J.M., GERBER, M.R. and 4 others (1987). Deletions of a DNA sequence in retinoblastomas and mesenchymal tumours: organization of the sequence and its encoded protein. *Proc. Natl Acad. Sci. USA*, **84**, 9059.

FRIEND, S.H., DRYJA, T.P. & WEINBERG, R.A., (1989). Oncogenes and tumour-suppressing genes. *N. Engl. J. Med.*, **318**, 618.

GENDLER, S.J., BURCHELL, J.M., DUHIG, T and 4 others (1987). Cloning of partial cDNA encoding differentiation and tumour-associated mucin-glycoproteins expressed by human mammary epithelium. *Proc. Natl Acad. Sci. USA*, **84**, 6060.

HARRIS, H. (1986). Malignant tumours generated by recessive mutations. *Nature*, **323**, 582.

JARMAN, A.P., NICHOLLS, R.D., WEATHERALL, D.J., CLEGG, J.B. & HIGGS, D.R. (1986). Molecular characterisation of a hypervariable region downstream of the human alpha-globin gene cluster. *EMBO J.*, **5**, 1857.

JEFFREYS, A.J., ROYLE, N.J., WILSON, V. & WONG, Z. (1988). Spontaneous mutation rates to new length alleles at tandem-repetitive hypervariable loci in human DNA. *Nature*, **332**, 278.

KNUDSON, A.G. JR (1985). Hereditary cancer, oncogenes and anti-oncogenes. *Cancer Res.*, **45**, 1473.

LAW, D.J., OLSCHWANG, S., MONPEZAT, J.P. and 5 others (1988). Concerted nonsynthetic allelic loss in human colorectal carcinoma. *Science*, **241**, 961.

MANIATIS, T., FRITSCH, E.F. & SAMBROOK, J. (1982). *Molecular Cloning. A Laboratory Manual.* Cold Spring Harbor Laboratory: New York.

MOTOMURA, K., NISHISHO, I., TAKAI, S.-I. and 6 others (1988). Loss of alleles at loci on chromosome 13 in human primary gastric cancers. *Genomics*, **2**, 180.

NAKAMURA, Y. LEPPERT, M., O'CONNELL, P. and 8 others (1987). Variable number of tandem repeat (VNTR) markers for human gene mapping. *Science*, **235**, 1616.

OKAMOTO, M., SASAKI, M., SUGIO, K. and 6 others (1988). Loss of constitutional heterozygosity in colon carcinoma from patients with familial polyposis coli. *Nature* **331**, 273.

SANDBERG, A.A. (1980). Solid tumours and metastatic cancer. Tumours of the alimentary tract. In *The Chromosomes in Human Cancer and Leukemia*, Sandberg, A.A. (ed) p. 468. Elsevier: New York, Amsterdam.

SOLOMON, E., VOSS, R., HALL, V. and 6 others (1987). Chromosome 5 loss in human colorectal carcinomas. *Nature*, **328**, 616.

SWALLOW, D.M., GENDLER, S., GRIFFITHS, B., CORNEY, G., TAYLOR-PAPADIMITRIOU, J. & BRAMWELL, M.E. (1987*a*). The human tumour-associated epithelial mucins are coded by an expressed hypervariable gene locus PUM. *Nature*, **328**, 82.

SWALLOW, D.M., GENDLER, S., GRIFFITHS, B. and 5 others (1987*b*). The hypervariable gene locus PUM, which codes for the tumour associated epithelial mucins, is located on chromosome 1, within the region 1q21–24. *Ann. Human Genet.*, **51**, 289.

VOGELSTEIN, B., FEARON, E., HAMILTON, S.R. and 7 others (1988). Genetic alterations during colorectal-tumor development. *N. Engl. J. Med.*, **319**, 525.

WADA, M., YOKOTA, J., MIZOGUCHI, H., SUGIMURA, T. & TERADA, M. (1988). Infrequent loss of chromosomal heterozygosity in human stomach cancer. *Cancer Res.*, **48**, 2988.

WONG, Z., WILSON, V., JEFFREYS, A.J. & THEIN, S.L. (1986). Cloning a selected fragment from a human DNA 'fingerprint': isolation of an extremely polymorphic minisatellite. *Nucl. Acids Res.*, **14**, 4605.

WONG, Z., WILSON, V., PATEL, I., POVEY, S. & JEFFREYS, A.J. (1987). Characterization of a panel of highly variable minisatellites cloned from human DNA. *Ann. Human Genet.*, **51**, 269.

YOKOTA, J., TSUNETSUGU-YOKOTA, Y., BATTIFORA, H., LE FEVRE, C. & CLINE, M.J. (1986). Alterations of myc, myb, and $ras^{Ha}$ proto-oncogenes in cancers are frequent and show clinical correlation. *Science*, **231**, 261.

YUNIS, J.J. (1983). The chromosomal basis of human neoplasia. *Science*, **221**, 227.

YUNIS, J.J., BRUNNING, R., HOWE, R.B. & LOBELL, M. (1984). High-resolution chromosomes as an independent prognostic indicator in adult acute nonlymphocytic leukemia. *N. Engl. J. Med.*, **311**, 812.